# Cost-Effectiveness Analysis of Artificial Intelligence-Driven Risk Stratification in Patients With Diabetic Kidney Disease in the US Veterans Population

**DOI:** 10.1016/j.xkme.2026.101261

**Published:** 2026-01-12

**Authors:** Jyotirmoy Sarker, Abdullah I. Abdelaziz, Jacob Crook, Richard E. Nelson, Joanne LaFleur, Heather Nyman, Chao-Chin Lu, Kibum Kim

**Affiliations:** 1 Department of Pharmacy Systems, Outcomes and Policy, University of Illinois Chicago, Chicago, IL; 2 Department of Pharmacotherapy, University of Utah, Salt Lake City, UT; 3 Salt Lake City VA Health Care System, Salt Lake City, UT; 4 Division of Epidemiology, University of Utah, Salt Lake City, UT

**Keywords:** Artificial intelligence, cost-effectiveness, diabetic kidney disease, DKD risk stratification, Veterans Health Administration

## Abstract

**Rationale & Objective:**

Efficient risk stratification is essential to optimize care and allocate resources for treatment of diabetic kidney disease (DKD). This study evaluates the cost-effectiveness of an artificial intelligence-driven in vitro kidney disease risk assay (AIKD).

**Study Design:**

Cost-effectiveness analysis using a hybrid model, combining a decision tree followed by a Markov model.

**Setting & Population:**

Patients with early-stage DKD receiving care within the US Veterans Health Administration health care system.

**Intervention(s):**

Risk stratification using AIKD versus Kidney Disease: Improving Global Outcomes (KDIGO) standard of care (SoC).

**Outcomes:**

Five-year health care costs, quality-adjusted life-years (QALYs), and incremental cost-effectiveness ratio (ICER).

**Model, Perspective, & Timeframe:**

The decision tree delineated clinical pathways based on the prevalence of progressive decline in kidney function and risk stratification performance of AIKD versus KDIGO. The subsequent Markov model simulated DKD stage transitions across the underlying risk–treatment pathways. Model inputs included test performance characteristics, risk prevalence, transition probabilities, costs, and utilities. One-way and probabilistic sensitivity analyses assessed uncertainty. The analysis was conducted from the perspective of the Veterans Health Administration health care system over a 5-year time horizon.

**Results:**

AIKD-guided care resulted in a total cost of $146,437 and 2.8277 QALYs, compared with $145,120 and 2.8164 QALYs for the SoC arm. The ICER for AIKD relative to SoC was $116,349 per QALY gained. One-way sensitivity analysis showed that the sensitivity and specificity of AIKD and SoC, as well as the prevalence of underlying risk of progressive decline in kidney function, were the most influential inputs affecting the ICER. From the probabilistic sensitivity analysis, AIKD has 69% likelihood of being accepted at the conventional willingness-to-pay threshold of $150,000 per QALY gained.

**Limitations:**

Model assumptions regarding risk stratification performance and long-term treatment effects may limit generalizability.

**Conclusions:**

AIKD is cost-effective compared to KDIGO for patients with early-stage DKD. Its adoption could improve health outcomes and support efficient health care resource utilization management.

## Plain-Language Summary

We studied whether a new artificial intelligence tool, artificial intelligence-driven in vitro kidney disease risk assay (AIKD), is a worthwhile investment for early identification of diabetic kidney disease compared with the current Kidney Disease: Improving Global Outcomes (KDIGO) guideline-recommended risk stratification method. This tool assists clinicians in identifying patients at high risk of kidney function decline, allowing for earlier and more targeted interventions. Using data from the US Veterans Health Administration, we found that AIKD modestly increases care costs but helps slow disease progression and improves quality of life. Over 5 years, AIKD was found to be cost-effective, and its overall impact on the health care budget was modest. Our findings suggest that AIKD-assisted personalized care decision making has the potential to result in cost-effective health care resource utilization in patients with diabetic kidney disease.

Chronic kidney disease (CKD) is a major public health concern, affecting approximately 14% of adults in the United States.^1^ In 2020, the health care expenditure for patients with CKD (excluding patients with kidney failure) was $85.4 billion, representing 23.5% of the total Medicare expenditure.^2^ For patients with kidney failure, the Medicare expenditure reached $50.8 billion.^2^

Diabetes is considered the risk factor for the onset of CKD, with 1 in 3 diabetic adults developing CKD.^3^^,^^4^ Patients with diabetic CKD (DKD) can experience an accelerated decline in kidney function due to hypertension and other metabolic disorders. Moreover, the health care costs associated with DKD were found to be substantially higher than those of patients with CKD without diabetes.^5^ With the prevalence of diabetes on the rise, DKD cases are expected to increase. Despite the fact that clinical and economic burdens are well recognized, DKD remains highly underdiagnosed, with only 10% of patients at minimal risk and 49% of those at high risk of kidney failure being aware of their condition.^6^ This prompted the American Diabetes Association and Kidney Disease: Improving Global Outcomes (KDIGO) to recommend annual screening for CKD in patients with diabetes.^7^

Accurate prediction of the rapid decline in kidney function can assist providers in planning treatment pathways, such as prescribing renal protective agents to delay DKD progression and to reduce the number of kidney failure cases. Recent advancements in computational sciences and novel biomarker screenings have paved the way for artificial intelligence-based approaches to exhibit significant potential in risk stratification.^8^, ^9^, ^10^ A recent example of this is an artificial intelligence-driven in vitro diagnostic assay for kidney disease (AIKD), which is used to assess the risk of kidney disease progression.^11^ AIKD received a de novo marketing authorization from the US Food and Drug Administration.^12^ The AIKD algorithm uses a supervised machine learning model, specifically a random forest algorithm. It integrates data for 3 plasma biomarkers—soluble tumor necrosis factor receptors 1 and 2 and kidney injury molecule-1—with clinical data from electronic medical records, such as serum urea nitrogen, hemoglobin A_1c_, and urinary albumin-creatinine ratio. The algorithm is applied to adults with type 2 diabetes and CKD stages G1 to G3b to stratify patients into 3 risk categories: high, medium, and low risk for 5-year progressive decline in kidney function (PDKF).^13^ In a performance validation study, the model achieved an area under the receiver operating characteristic curve of 0.77 (95% confidence interval, 0.76-0.79), outperforming a standard clinical model (area under the curve, 0.61; 95% confidence interval, 0.60-0.63).^14^ The positive predictive value in the high-risk group reached 61%, while the negative predictive value in the low-risk group was 90%, exceeding the performance of the standard KDIGO risk stratification method (positive predictive value, 40%).^14^ Once properly implemented, AIKD can assist providers in referring high-risk DKD patients to specialty care or in making treatment decisions for switching to renal protective medications.^15^^,^^16^

The burden of DKD is particularly significant in the US Veterans Health Administration (VHA) health system. Veterans are 34% more likely to have kidney disease compared with the general population, which is mainly attributable to the higher prevalence of diabetes among veterans.^17^^,^^18^ In addition, DKD progresses faster among veterans compared to the other DKD patients in the US population.^18^ Thus, the VHA health system can benefit from implementing advanced decision-supporting tools like AIKD to maximize clinical benefits and reduce long-term costs. In a recent budget impact model, Datar et al^11^ showed that implementing AIKD can reduce health care costs by $145 million in 5 years. However, the previous model did not consider the specific impact of the test’s performance, potentially undermining the validity of the projection. Moreover, Datar et al^11^ derived model inputs using population-based estimates from the literature, which raises concerns about the applicability of the model’s results to a unique health care system such as the VHA.^19^^,^^20^ To address this gap, an evaluation framework that incorporates real-world clinical and cost inputs specific to the VHA system is needed. Therefore, we aimed to develop a cost-effectiveness and budget impact model for AIKD-directed risk stratification and medication decision making in patients with early-stage DKD within the VHA health system.

## Methods

### Overview

We estimated the cost-effectiveness of AIKD to assist the selection of treatment pathways in US veterans with DKD compared with KDIGO risk stratification. Under the KDIGO strategy, primary care physicians use patients’ health care records to make decisions about switching to an aggressive and comprehensive treatment. Both AIKD and the KDIGO strategy were assumed to be used in early-stage DKD patients (stages G1 to G3b or estimated glomerular filtration rate [eGFR] ≥30 mL/min/1.73 m^2^) to classify them into 1 of 3 risk categories: high, moderate, and low risk of developing PDKF within 5 years.^21^ PDKF was defined as an eGFR slope decline ≥5 mL/min/y, a sustained decline in eGFR ≥40% from baseline (confirmed after ≥3 months), or the occurrence of kidney failure (defined as sustained eGFR <15 mL/min or long-term maintenance dialysis or kidney transplantation [KT]).^13^^,^^21^ Patients who were categorized as high-risk for PDKF by either AIKD or KDIGO risk stratification will receive comprehensive DKD management consisting of therapy modifications (replacing previously used antidiabetic agents with a renal protective sodium/glucose cotransporter 2 inhibitor along with an angiotensin-converting enzyme inhibitor), annual diet counseling, and DKD management education.^11^^,^^22^ A recent validation study demonstrated that both the sensitivity and specificity of AIKD (51% and 93%) were superior to the performance of KDIGO risk stratification (28% and 88%).^14^ The greater improvement in sensitivity, relative to the specificity gain, allows more patients to receive comprehensive management after AIKD versus the KDIGO strategy. Hence, the ultimate goal of this cost-effectiveness study is to assess if the extra expenses for AIKD of $1,050 and comprehensive care are fully or partially offset by the delay in DKD progression and effectiveness gains measured in quality-adjusted life-years (QALYs). Analysis was conducted from the US VHA health care system perspective. We applied an annual discount rate of 3% for both costs and QALYs. Of note, the cost of the AIKD was determined based on direct communications with the service provider and a published study. Per a previous publication, the total cost was determined based on the $950 payment assigned in the Medicare fee schedule and an additional administrative cost of $100.^11^

### Model Structure and Clinical Pathways

A de novo decision tree was developed using Microsoft Excel. We assumed that patients would receive comprehensive DKD management or conventional care based on PDKF classification. Available data did not sufficiently address uncertainty around outcomes over an extended time horizon. Further, considering the dynamics and advance of the treatment strategies, patients will have to be regularly screened with AIKD, once a health care system has decided to cover the service. We projected the outcomes over the 5-year horizon that would match the interest of health policy and decision makers.

The simulated cohort is divided into 2 groups based on projected outcomes over 5 years in the absence of comprehensive care: patients who are expected to develop PDKF and those who are not. To account for test accuracy, the decision tree further divides the cohort into 4 subgroups: true positives, which includes patients who will develop PDKF within 5 years and are correctly classified by the test as high risk; false negatives, which includes patients who will develop PDKF within 5 years but are incorrectly classified as low or moderate risk; false positives, which includes patients who will not develop PDKF within 5 years but are incorrectly classified as high risk; and true negatives, which includes patients who will not develop PDKF within 5 years and are correctly classified as low or moderate risk. In both groups (AIKD and KDIGO), patients with high predicted PDKF risk (true positives and false positives) receive comprehensive care. In contrast, true negative and false-negative patients continue to receive conventional management with fewer renal protective antidiabetic agents.

**Figure 1:**
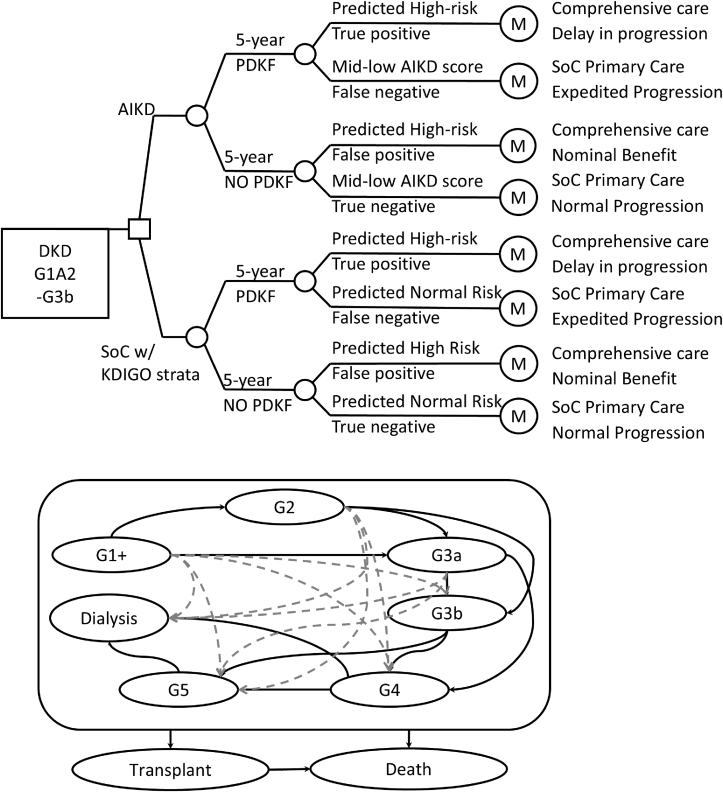
Decision tree and Markov model. From the base case simulation, 18% under AIKD and 16% under Kidney Disease: Improving Global Outcomes (KDIGO) receive comprehensive care at the end of year 1. Abbreviations: AIKD, artificial intelligence-driven in vitro kidney disease risk assay; DKD, diabetic kidney disease; PDKF, progressive decline in kidney function; SoC, standard of care.

The clinical decision pathway is followed by a Markov model in which patients start from one of the early DKD stages (stages G1-G3b) and advance to any of the model states based on their PDKF status (Fig 1). Patients could stay in the same stage or progress to an advanced stage at the end of each 1-year cycle, but transitioning to a less severe stage is not allowed given the progressive nature of DKD. Patients can also move directly from any state in the model to a transient KT state or the terminal “Death” state (Fig 1). The transient KT state addresses the acute care cost and utility of the transplant surgery and intensive care for the first 3 months after KT, which could be followed by either post-KT or Death state.

### Target Population, Epidemiology, and Clinical Inputs

The cohort consisted of early-stage DKD patients across stages G1, G2, G3a, and G3b, with respective proportions of 2.8%, 13.9%, 68.9%, and 14.4%. The mean age was 65 years, and 96.3% of the cohort were male. These characteristics were consistent with those observed in our prior epidemiologic analysis of the VHA population.^23^ The DKD progression probabilities were derived from the same epidemiologic study and are presented in Table 1.^14^^,^^24^, ^25^, ^26^, ^27^

**Table 1:** Model Inputs

Input	Base Case	Plausible Range (Used for OWSA)	PSA Distribution	Source^a^
General inputs				
Age, y	65	50-70	—	VHA epidemiology study
Cycle length, y	1	—	—	—
Time horizon, y	5	—	—	—
Discount rate, %	3%	—	—	—
Decision tree inputs				
Test performance parameters				
AIKD sensitivity, %	51%	41%-61%	Beta	Chan et al^14^ (2021)
AIKD specificity, %	93%	66%-100%	Beta	
Standard of care sensitivity, %	28%	23%-34%	Beta	
Standard of care specificity, %	88%	66%-100%	Beta	
Prevalence of DKD patients with true PDKF by stage				
G1	0.617	0.11-0.31	Beta	VHA epidemiology study
G2	0.610	0.11-0.30	Beta	
G3a	0.794	0.16-0.36	Beta	
G3b	0.815	0.17-0.37	Beta	
Overall	0.766	0.60-0.90	Beta	
Cost of KidneyIntelX	$1,050	$854-$1,266	Gamma	
Markov chains inputs				
Prevalence of DKD stages, %				
G1	2.8%	2.8%-2.9%	Beta	VHA epidemiology study
G2	13.9%	13.8%-14.0%	Beta	
G3a	68.9%	68.7%-69.0%	Beta	
G3b	14.4%	—	—	
Annual rates of DKD stages progression to any advanced stage or death among non-PDKF patients				
G1	0.110	0.106-0.112	Beta	VHA epidemiology study
G2	0.088	0.086-0.089	Beta	
G3a	0.105	0.104-0.106	Beta	
G3b	0.150	0.148-0.151	Beta	
G4	0.242	0.238-0.246	Beta	
G5	0.326	0.316-0.337	Beta	
Long-term dialysis	0.222	0.216-0.228	Beta	
Kidney transplant	0.049	0.034-0.045	Beta	
Postkidney transplant	0.077	0.059-0.071	Beta	
The effect of comprehensive care on progression by DKD stage^b^				
G1 (true positive)	0.37	0.28-0.46	Normal	Neuen et al^24^ (2019)
G2 (true positive)	0.60	0.45-0.75	Normal	
G3a (true positive)	0.55	0.45-0.75	Normal	
G3b (true positive)	0.70	0.45-0.75	Normal	
Any stage (false positive)	0.95	0.90-1.00	Normal	Assumption
The impact of PDKF status on progression^c^				
G1	1.671	1.651-1.690	Normal	VHA epidemiology study
G2	1.986	1.979-1.993	Normal	
G3a	1.579	1.577-1.582	Normal	
G3b	1.315	1.310-1.320	Normal	
Cost^d^, USD				
G1	$19,164	$15,593-$23,098	Gamma	VHA cost study
G2	$21,264	$17,031-$25,629	Gamma	
G3a	$34,284	$27,895-$41,322	Gamma	
G3b	$44,664	$36,340-$53,833	Gamma	
G4	$66,060	$53,749-$79,621	Gamma	
G5	$83,988	$68,336-$101,230	Gamma	
Long-term dialysis	$147,576	$120,074-$177,872	Gamma	
Kidney transplant	$71,958	$123,340-$182,710	Gamma	
Postkidney transplant	$79,632	$64,792-$95,980	Gamma	
Cost of comprehensive care (SGLT2i replacement + additional primary or nephrology visits)	$2,655	$1,991.25-$3,318.75	Gamma	
CKD/DKD education	$143^e^	$108-$179	Uniform	
Health utility and disutility				
Population utility constant	0.944	0.892-0.997	Beta	Sullivan and Ghushchyan^25^ (2006)
Utility decrement per age	0.0007	0.0006-0.0008	Beta	
Disutility at G1	0.150	0.121-0.180	Beta	Cooper et al^26^ (2020); Kennedy-Martin et al^27^ (2015)
Disutility at G2	0.150	0.121-0.180	Beta	
Disutility at G3a	0.200	0.162-0.241	Beta	
Disutility at G3b	0.200	0.162-0.241	Beta	
Disutility at G4	0.260	0.211-0.313	Beta	
Disutility at G5	0.270	0.219-0.325	Beta	
Disutility at long-term dialysis	0.530	0.426-0.633	Beta	
Disutility at kidney transplant	0.530	0.426-0.633	Beta	
Disutility at postkidney transplant	0.290	0.235-0.348	Beta	

Abbreviations: AIKD, artificial intelligence-driven in vitro kidney disease risk assay; CKD, chronic kidney disease; DKD, diabetic kidney disease; OWSA, one-way sensitivity analysis; PDKF, progressive decline in kidney function; PSA, probabilistic sensitivity analysis; SGLT2i, sodium/glucose cotransporter 2 inhibitor; USD, United States dollars; VHA, Veterans Health Administration.

a Source for base-case inputs.

b Reflected by the relative risk of DKD stage progression comparing the risk of progression among PDKF patients receiving comprehensive care versus that of patients that are not receiving comprehensive care.

c Reflected by the relative risk of DKD stage progression comparing the risk of progression among PDKF patients versus that of patients without PDKF.

d Annual, unless specified.

e This consists of dietitian visit ($32.53) and CKD education ($109.46).

To reflect the differential rate of progression following each of the treatment pathways, we performed a post hoc analysis of the same VHA cohort and calculated the stage progression rate stratified by the presence of 5-year PDKF from the index stages G1-G3b. We estimated the relative risk of stage progression in the PDKF group compared with the no-PDKF group. The corresponding values are presented in Table 1. Once it progressed to stage G4 or above, PDKF would not be considered as a clinical or predictive marker of interest, and renal protective care would become a part of standard management to protect patients from progression to kidney failure.^13^^,^^21^ Also, we defined the state transition probability inputs for stages G4, G5, KT, and post-KT directly from our analysis and the literature.^28^, ^29^, ^30^, ^31^

Comprehensive care is anticipated to decrease the rate of progression, particularly related to tubuloglomerular feedback and the intraglomerular pressure effect of renal protective agents.^32^^,^^33^ We abstracted the DKD stage progression hazard ratio for the renal protective agents, ranging between 0.37 and 0.70, from recent clinical trials assessing the clinical outcomes of sodium/glucose cotransporter 2 inhibitors.^24^ In our simulation, such a large benefit was applied to the true positive arm only to account for the clinical benefits of comprehensive care in patients with increased risk of progression, whereas a nominal benefit was applied for the patients at a normal risk of PDKF who receive comprehensive care. The assumed nominal benefits for the false positives versus true negatives was addressed by calculating a hazard ratio for the stage progression of 0.95 for comprehensive care versus conventional care. We made the assumption that the delay in DKD progression for patients in the true positive pathway that was attributable to the clinical benefits of comprehensive management would not exceed the rate of progression among patients in the true negative pathway. To address this point, our model limits the maximum benefits of correctly administered comprehensive care by the inverse of the relative risk of the rate of progression for the PDKF versus no-PDKF group, which was calculated from the post hoc analysis of the DKD patient cohort (Fig S1).

### Health Utility and Cost

The primary measure of effectiveness was QALY, estimated by summing, over all cycles, the product of the state-specific utility, the proportion of the cohort in each health state, and the cycle length. We used an age-specific fixed baseline utility value that diminishes over time by a constant coefficient.^25^ The disutility of each DKD stage and KT were identified from the literature and then applied to the age-specific general utility to calculate the state-specific utility score.^26^^,^^27^

We identified cost inputs from a recent analysis of DKD cost in the VHA population and a review of publicly available databases.^34^ Briefly, total health care cost per unit period in veterans with DKD, from the VHA perspective, monotonically increases with stage progressions from stages G1 to G5, and monthly care cost exceeds $10,000 once the patient receives long-term dialysis.^34^ Replacing sulfonylurea or dipeptidyl peptidase 4 inhibitors with sodium/glucose cotransporter 2 inhibitors is the primary component of comprehensive care. The cost associated with such replacement was defined from the VA Federal Supply Schedule table.^35^ It is associated with a $2,655 annual cost escalation for our model. Costs of 2 additional primary care or nephrology visits per cycle for comprehensive care were considered within this category. We also included the once-a-year dietitian visit ($32.53) and CKD education ($109.46) in calculating the cost of comprehensive care.^36^

### Analysis and Simulation

The relative value of AIKD versus the KDIGO method was presented in the incremental cost-effectiveness ratio (ICER) calculated from the increment in discounted cost per unit QALY gained. We compared the ICER estimate with the generally acceptable and liberal willingness-to-pay (WTP) thresholds of $150,000 and $200,000 per QALY, respectively.

The robustness of our model and conclusion were evaluated using various sensitivity analyses. For one-way sensitivity analysis, a single model input was varied within the 95% confidence interval range for each simulation while all other model inputs were fixed at the base case. When the standard error for the confidence interval calculation was unavailable, we used the ±25% range of the base-case input for the one-way sensitivity analysis. We used a tornado diagram to present the ICER ranges from the one-way sensitivity analysis.

The overall probability of AIKD being cost-effective at varying levels of WTP thresholds was tested using a probabilistic sensitivity analysis, for which we simultaneously varied all model inputs with specific distributional assumptions (Table 1). Results from 1,000 Monte-Carlo simulations were presented both as a cost-effectiveness plane and as a cost-effectiveness acceptability curve for the WTP threshold range of $0-$200,000 per QALY gained.

### Scenario Analysis

We conducted 2 scenario analyses. In the first scenario analysis, patients classified as a moderate risk were allowed to receive comprehensive care. This scenario assessed the trade-off of higher sensitivity and lower specificity of both AIKD and the KDIGO strategy in treatment decisions. For the second scenario analysis, we replaced the proportion of patients having prospective PDKF with 50% for all the initial DKD stages, which approximately doubled the true risk of rapid progression. In this scenario, we focused on high-risk patient cohorts, which likely decreased the ICER for AIKD compared with KDIGO risk stratification.

### Budget Impact Analysis

Using the cost-effectiveness model, we estimated the potential budgetary impact of AIKD for VHA. Based on our analysis of the VHA epidemiology data, the anticipated number of patients eligible for AIKD is approximately 420,000.^28^ We assumed that 10% of the eligible subjects (n = 42,000) would receive AIKD and assessed the 5-year budget impact of the 42,000 AIKD-based risk stratification. A second budget impact analysis tested the scenario that the same number of tests will be continuously offered over the 5-year period (ie, a total of 240,000 tests equally distributed over the 5 years) for the veterans who are naïve to the AIKD. The cumulative budget impact over the 5-calendar year period was calculated using the difference in the undiscounted costs.

### Ethics Approval

The cost-effectiveness analysis and modeling study was limited to literature reviews, evidence synthesis, and calculation without involving human subjects or subject-level data. Thus, this study does not qualify for institutional review board review.

## Results

### Base-Case Analysis

While KDIGO risk stratification costs $145,120 for 2.8164 QALYs, the AIKD-based stratification incurs a higher expense of $146,437 and results in 2.8277 QALYs over the 5-year time horizon. This represents an additional expenditure of $1,317 for an incremental effectiveness of 0.0113 QALY gained, resulting in an ICER of $116,349 per QALY gained (Table 2).

**Table 2:** Base Case and Scenario Analyses

	AIKD		KDIGO		ICER ($/QALY)
	Discounted Cost	Discounted QALY	Discounted Cost	Discounted QALY	
Base case	$146,437	2.8277	$145,120	2.8164	$116,349
Liberal decision	$150,902	2.8504	$148,812	2.8396	$192,930
High-risk scenario	$148,478	2.7218	$145,466	2.6864	$85,130

*Note:* Liberal decision scenario: Patients classified as either moderate or high risk of PDKF would receive comprehensive care. High-risk scenario: 50% of the target cohort with DKD stages G1-G3b will eventually develop PDKF if additional comprehensive care is not given.

Abbreviations: AIKD, artificial intelligence-driven in vitro kidney disease risk assay; DKD, diabetic kidney disease; ICER, incremental cost-effectiveness ratio; KDIGO, Kidney Disease: Improving Global Outcomes; PDKF, progressive decline in kidney function; QALY, quality-adjusted life-year.

### Sensitivity Analysis

**Figure 2:**
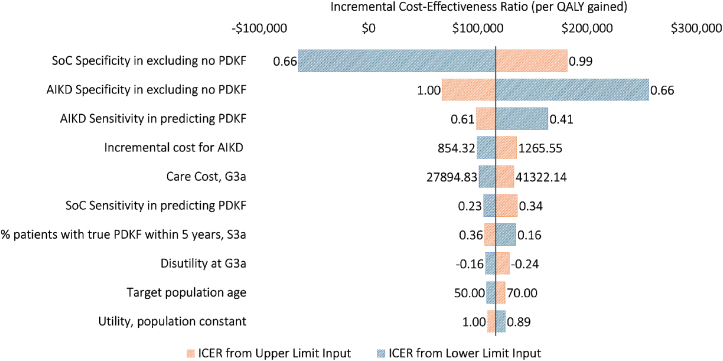
Tornado diagram from one-way sensitivity analysis. ICER from lower and upper input limits. Abbreviations: AIKD, artificial intelligence-driven in vitro kidney disease risk assay; ICER, incremental cost-effectiveness ratio; PDKF, progressive decline in kidney function; QALY, quality-adjusted life-year; SoC, standard of care.

In the one-way sensitivity analysis, the most influential factors affecting the cost-effectiveness decision was the ability of AIKD and the KDIGO strategy to correctly exclude patients with no prospective risk of PDKF from the comprehensive care pathway. If AIKD is able to exclude patients from the comprehensive care route perfectly (100% specificity), ICER dropped to $69,214 per QALY gained. Other variables that significantly impact the ICER are sensitivity of AIKD, cost of AIKD, care cost for DKD stage G3a, and sensitivity of KDIGO risk stratification. No variables, except for the specificity of AIKD, resulted in the ICER exceeding the liberal WTP threshold of $200,000 per QALY gained (Fig 2).

**Figure 3:**
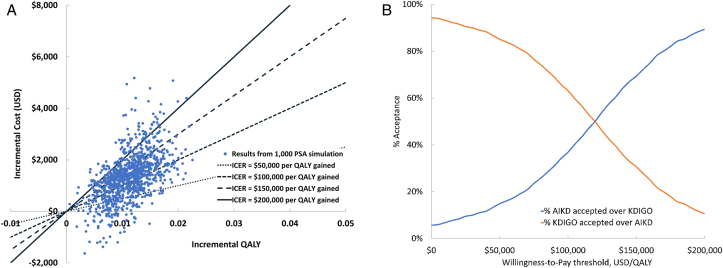
Results from probabilistic sensitivity analysis. (A) Scatter plot of the incremental costs and QALYs. (B) Cost-effectiveness acceptability curves. Abbreviations: AIKD, artificial intelligence-driven in vitro kidney disease risk assay; ICER, incremental cost-effectiveness ratio; KDIGO, Kidney Disease: Improving Global Outcomes; PSA, probabilistic sensitivity analysis; QALY, quality-adjusted life-year; USD, United States dollars.

Results from the probabilistic sensitivity analysis demonstrated that AIKD was cost-effective compared to the KDIGO strategy in 69% of the simulations at a WTP threshold of $150,000 per QALY gained. The likelihood of AIKD being a cost-effective risk stratification strategy was 89% at a liberal WTP threshold of $200,000 per QALY gained (Fig 3A, B).

### Scenario Analysis Results

Compared with the base-case scenario where only high-risk patients receive comprehensive care management, the ICER rose to $192,930 per QALY gained under the scenario of allowing the moderate risk group to receive comprehensive care management. In the high-risk group scenario where half of the target population has prospective PDKF, AIKD-based treatment selection had a higher value with an estimated ICER of $85,130 per QALY gained (Table 2).

### Budget Impact Analysis

Implementing AIKD in the VHA for 42,000 patients has a total budget impact of $56 million over 5 years. The first-year impact of $46,718,000 was followed by the respective annual downstream effects of $2,633,000, $2,436,000, $2,183,000, and $1,969,000 (Fig S2). If AIKD is implemented in the same number of newly diagnosed patients each year over a 5-year period within the VHA, the aggregated budget impact is projected to be $258 million (Fig S3). The aggregated budget impact was approximately 0.07% of the total VHA budget, assuming the annual budget stays at the current level of $70 billion.

## Discussion

In this analysis, AIKD was associated with higher costs and greater QALY gains compared with conventional KDIGO risk stratification, making AIKD a cost-effective option at a WTP threshold of $150,000 per QALY. Although the implementation of AIKD does not translate into overall budget savings, moderate impacts on gross health care expenses would be offset by gained QALYs.

Economic outcomes of screening strategies for patients with kidney disorders have interested health economics and outcomes researchers. For example, Yarnoff et al^37^ assessed the cost-effectiveness of identifying patients at high risk for early-stage kidney screening. Using a CKD policy model consisting of 7 distinct CKD stages, the study demonstrated the cost-effectiveness of identifying a broader population for CKD screening with testing for albuminuria.^37^, ^38^, ^39^ A simplified progression model was also used to assess the cost-effectiveness of genotype-based treatment response prediction in patients with CKD. Those studies showed the value of pharmacogenetic screening to assist providers in making informed angiotensin-converting enzyme inhibitor use decisions for patients with early-stage estimated glomerular filtration rate decline.^40^^,^^41^ Recently, Datar et al^11^ calculated the payer budget impact of AIKD, which resulted in the estimated 5-year budget impact of $1.052 billion in savings per 100,000 DKD patients tested. The large savings were primarily attributable to an ideal scenario in which the clinical benefits of AIKD significantly reduced the stage progression rate by 15% among all screened patients.^11^ Building on this body of evidence, our study is unique in evaluating the cost-effectiveness of an artificial intelligence-driven screening intervention within the VHA, the largest integrated health care system in the United States. Unlike prior models, we directly sourced our clinical and economic model inputs using real-world evidence from the VHA database, enhancing both the internal validity and contextual relevance of our findings.

The findings of this study should be interpreted cautiously in light of several limitations. First, we directly abstracted the main clinical and cost inputs from studies on veterans with DKD and conducted the analysis from the VHA health care system’s perspective. Therefore, any costs incurred or reimbursed outside the VHA system are beyond the scope of our research. Since general DKD patients typically have lower baseline disease risk profiles than veterans with DKD and are covered under various health care reimbursement models, our ICER estimates would not directly apply to the general population or health care sectors other than the VHA system.^17^^,^^18^^,^^42^ Second, artificial intelligence and machine learning involve continuous computational training processes and are expected to improve over time as they are exposed to newer health care data. Consequently, the future performance of AIKD will depend on ongoing model retraining with additional data, and potential changes in sensitivity and specificity may, in turn, influence subsequent ICER estimates.^43^^,^^44^ Furthermore, the cost of AIKD will likely change after large-scale implementation due to economies of scale, and that can reduce the intervention cost.^45^^,^^46^ Therefore, our current estimates should be revisited and updated as new evidence emerges following the real-world implementation of AIKD.

Beyond limitations related to risk stratification strategies and target population, our model may not fully capture the heterogeneity of treatment scenarios, including therapy switching, early discontinuation of renal protective agents, or alternative treatment pathways for patients with contraindications. These limitations should be considered when interpreting our findings in the context of decision making for high-risk populations.^47^, ^48^, ^49^ Another limitation of the study is that we did not compare the cost-effectiveness of AIKD against any specific existing risk stratification methods. Although this analysis focused on the use and cost-effectiveness of AIKD for risk stratification at stages G1 to G3b, other models such as the Kidney Failure Risk Equation,^50^^,^^51^ the Kaiser Permanente Northwest equation,^52^ the Z6 score,^53^ and the model developed by Landray et al^54^ are validated tools primarily designed to predict kidney failure in patients with more advanced CKD, specifically stages G3 to G5. There are also other recently developed prediction and stratification models, such as Klinrisk,^55^ and the model developed by the Chronic Kidney Disease Prognosis Consortium,^56^ which are indicated for risk classification at earlier stages of CKD. Future research should compare the risk prediction accuracy and cost-effectiveness of AIKD both as a standalone stratification tool and as a supplement to existing models, considering the stage of disease and clinical context. Finally, any general limitation of the model-based approach, including subjectivity to the quality of inputs, various assumptions that cannot be tested in real-world settings, and debates around using discounted QALYs as a clinical outcome measure, should always be carefully considered.^57^, ^58^, ^59^

## Conclusions

Our analysis found that implementing AIKD in VHA settings to assist primary care providers in making preemptive decisions to switch to comprehensive care management based on PDKF predictions is cost-effective compared to the standard KDIGO risk stratification. The performance of risk stratification is the most critical input in the cost-effectiveness decision. As the predictive accuracy of the AIKD model may improve with continued training on updated datasets, future research should focus on validating its clinical utility, adaptability, and cost-effectiveness across diverse DKD populations.

## Footnotes

Supplementary File (PDF)
**Figure S1:** Cumulative incidence of progression stratified by PDKF. **Figure S2:** Five-year downstream budget impact of a one-year AIKD implementation to 42,000 patients. **Figure S3:** Five-year budget impact of AIKD implementation to 42,000 patients annually over the five years.
