# Supplementary material for: Cost-Effectiveness Analysis of Artificial Intelligence-Driven Risk Stratification in Patients With Diabetic Kidney Disease in the US Veterans Population
Source: Kidney Med. 2026 Jan 12;8(3):101261. doi: 10.1016/j.xkme.2026.101261 (PMC13080567; doi:10.1016/j.xkme.2026.101261)

Figure S1. Cumulative incidence of progression stratified by PDKF

From Stage 1

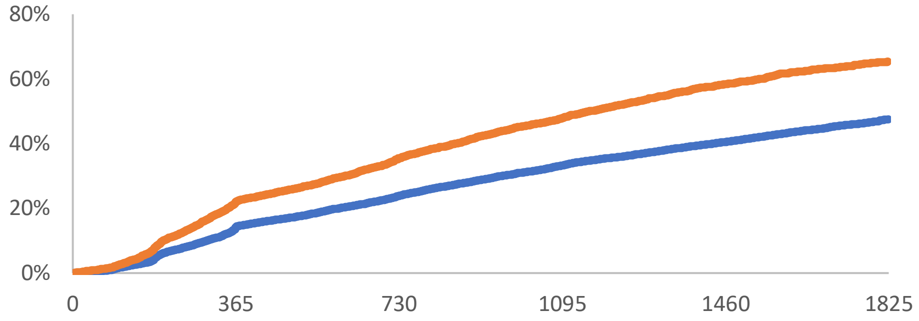

Day(s)	*365*	*730*	*1095*	*1460*	*1825*
PDKF	*22.18%*	*35.49%*	*47.91%*	*58.42%*	*65.40%*
No PDKF	*14.31%*	*23.86%*	*33.31%*	*40.55%*	*47.55%*

From Stage 2

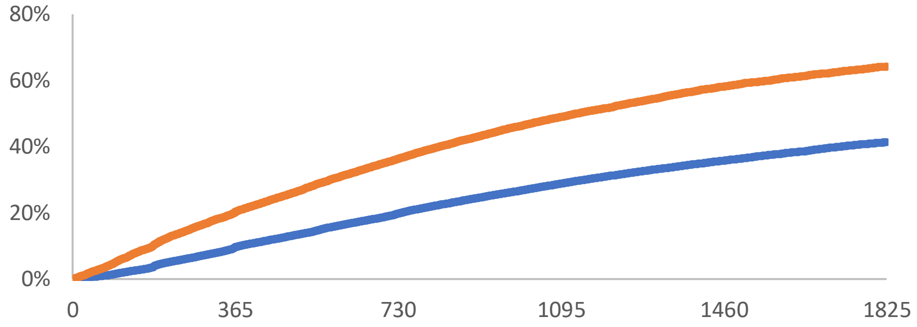

Day(s)	*365*	*730*	*1095*	*1460*	*1825*
PDKF	*20.38%*	*36.36%*	*48.96%*	*58.16%*	*64.20%*
No PDKF	*19.81%*	*28.83%*	*35.78%*	*41.36%*	*19.81%*

From Stage 3a

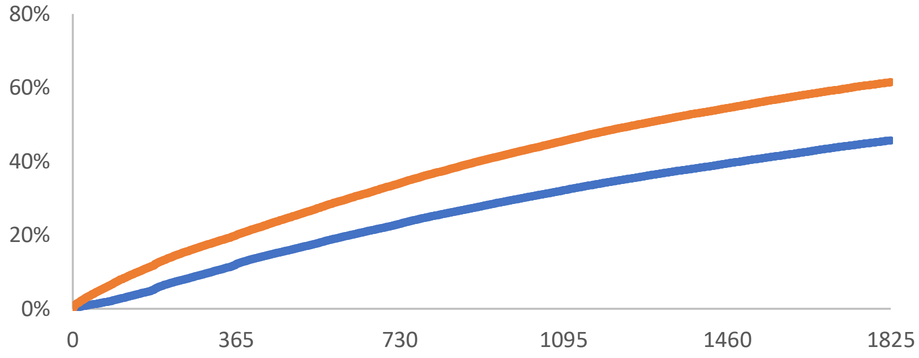

Day(s)	*365*	*730*	*1095*	*1460*	*1825*
PDKF	*20.05%*	*34.11%*	*45.57%*	*54.46%*	*61.47%*
No PDKF	*12.15%*	*23.09%*	*32.15%*	*39.43%*	*45.69%*

From Stage 3b

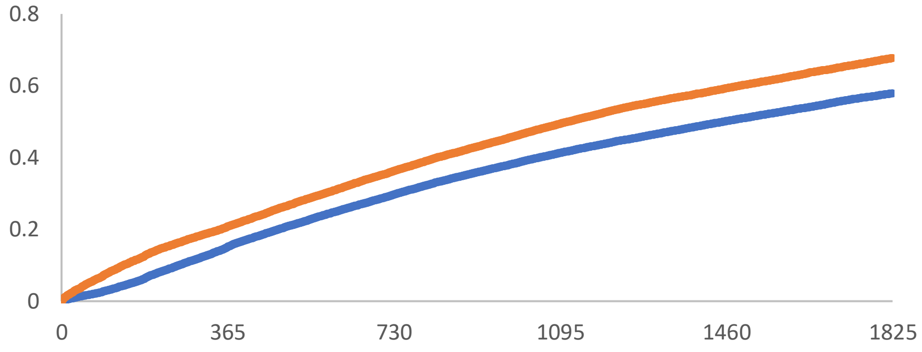

Day(s)	*365*	*730*	*1095*	*1460*	*1825*
PDKF	*20.82%*	*36.25%*	*49.44%*	*59.33%*	*67.73%*
No PDKF	*15.18%*	*29.70%*	*41.33%*	*50.18%*	*57.89%*

Y axis: cumulative incidence of DKD stage transition or death; X axis: number of days from the cohort entry; Orange: cumulative incidence estimates among patients with 5-year PDKF; Blue: cumulative incidence estimates among patients without 5-year PDKF.

Figure S2. Five-year downstream budget impact of a one-year AIKD implementation to 42,000 patients

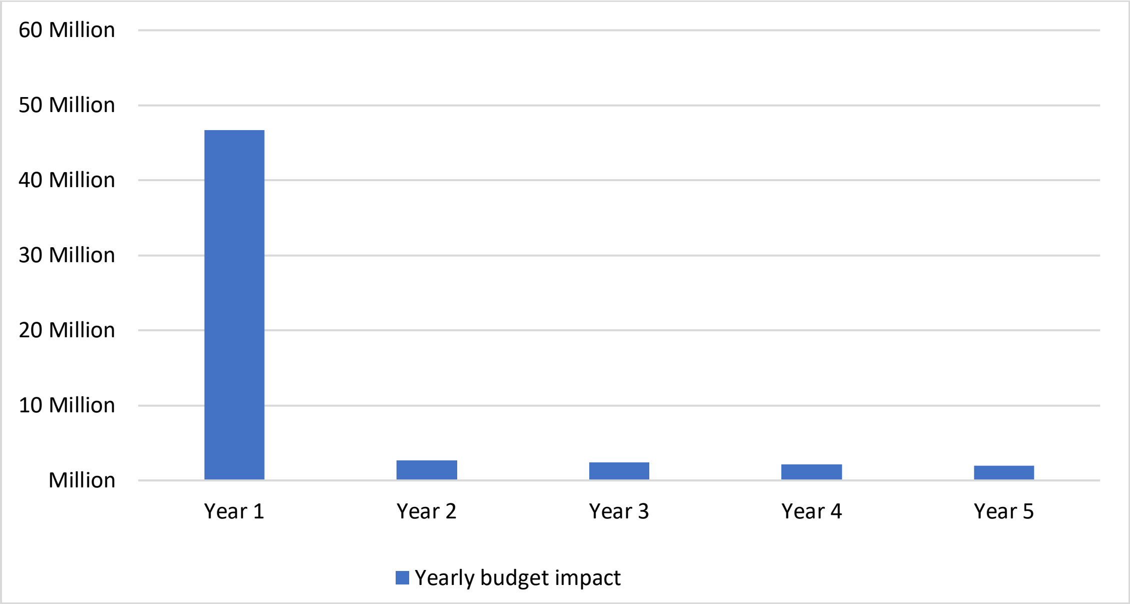

Figure S3. Five-year budget impact of AIKD implementation to 42,000 patients annually over the five years

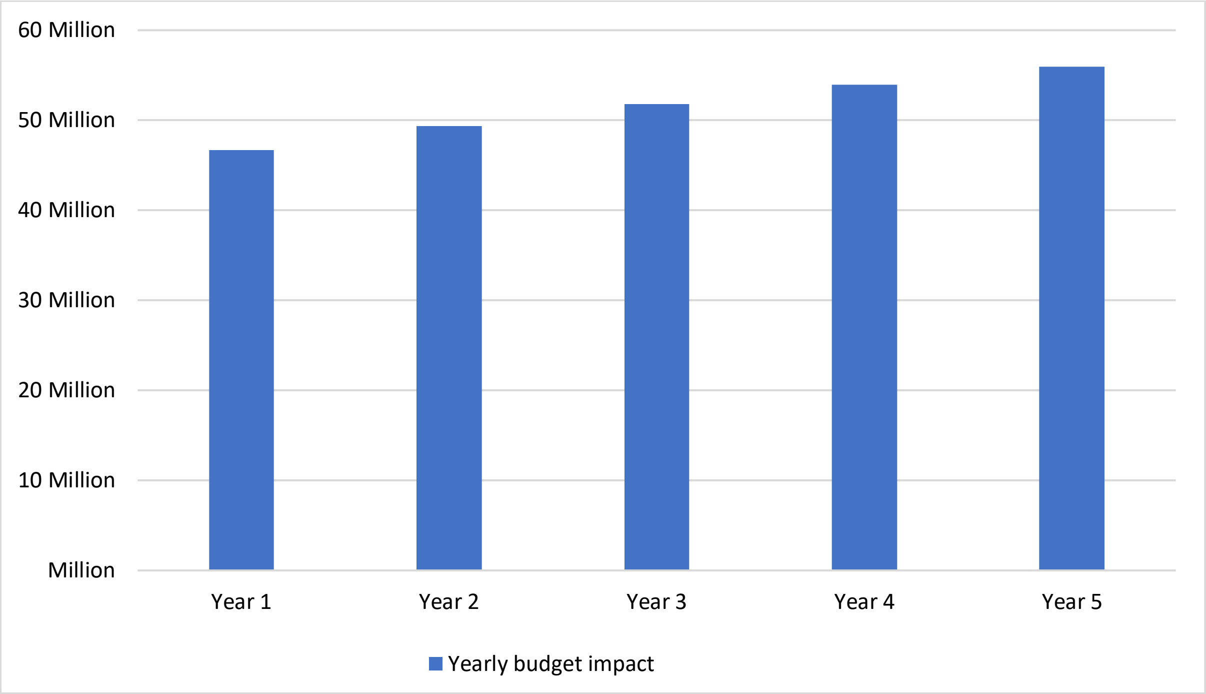

Supplement: Supplementary File (PDF) — Figures S1-S3. [file mmc1.pdf]
